# Prevention of Vitamin D deficiency in infancy: daily 400 IU vitamin D is sufficient

**DOI:** 10.1186/1687-9856-2011-4

**Published:** 2011-06-28

**Authors:** Gul Yesiltepe Mutlu, Yusuf Kusdal, Elif Ozsu, Filiz M Cizmecioglu, Sukru Hatun

**Affiliations:** 1 Department of Pediatrics, Division of Pediatric Endocrinology and Diabetes, Medical Faculty, Kocaeli University, Kocaeli, Turkey

## Abstract

**Summary:**

**Aim-objective**

Vitamin D deficiency and rickets in developing countries continues to be a major health problem. Additionally, the increase of cases of rickets in children of some ethnic groups in the United States and European countries has provided this issue to be updated. Obviously, powerful strategies are necessary to prevent vitamin D deficiency nation-wide. In 2005, a nationwide prevention program for vitamin D deficiency was initiated, recommending 400 IU vitamin D per a day.

This study was designed to evaluate the efficacy of the prevention program.

**Methods**

Eighty-five infants who were recalled as part of the national screening program for congenital hypothyroidism between February 2010 and August 2010 at Kocaeli University Children's Hospital were evaluated in terms of their vitamin D status as well. All babies had been provided with free vitamin D (Cholecalciferol) solution and recommended to receive 400 IU (3 drops) daily. Information regarding the age at start of supplementation, the dosage and compliance were obtained from the mothers with face-to-face interview. Serum 25-hydroxy vitamin D (25-OH-D), alkaline phosphatase (AP), parathormone (PTH) levels were measured.

**Results**

The mean age at which Vitamin D3 supplementation began was 16.5 ± 20.7 (3-120) days. Ninety percent of cases (n:76) were receiving 3 drops (400 IU) vitamin D3 per day as recommended; 70% of cases (n:59) were given vitamin D3 regularly, the remainder had imperfect compliance. Among those children who are older than 12 months, only 20% continued vitamin D supplementation. No subject had clinical signs of rickets. The mean 25-OH-D level was 42,5 ± 25,8 (median: 38.3) ng/ml. Ten subjects (12%) had their serum 25-OH-D levels lower than 20 ng/ml (6 between 15-20 ng/ml, 3 between 5-15 ng/ml and only one < 5 ng/ml).

**Conclusions**

400 U/day vitamin D seems adequate to prevent vitamin D deficiency. However, we believe that the program for preventing vitamin D deficiency in Turkey, needs to be reinforced to start immediately after birth, and to continue beyond 1 year of age at 400U regular daily dosage.

## Introduction

Vitamin D deficiency rickets has been a common child health problem in our sunny country with a recently reported incidence of 6-7% among children at age 0-3 years (1,2). Powerful strategies are necessary to prevent vitamin D deficiency nation-wide, because of this high incidence a prevention program to prevent vitamin D deficiency rickets was initiated across the country (3,4). Major obstacles in providing vitamin D supplementation in infants are limited public awareness, the cost of supplementation and limited access to healthcare. In May 2005, a national program (5-year project) was initiated to overcome those problems. In addition to, a nationwide campaign to encourage the entire population, particularly pregnant and nursing women and infants, to have adequate sunlight exposure and a curriculum to train healthcare workers, distribution of vitamin D supplements to every newborn was started throughout infancy at no financial cost to families through its network of primary care units and maternal-child health centers. The recommended dose was 400 IU (4). This campaign resulted in a dramatic decrease in the incidence of vitamin D deficiency rickets in some regions (5). However, there is inadequate data relating about the administration and effects on vitamin D levels.

This study was designed to gain insight about the efficacy of the prevention program locally in our region.

## Methods

The study was conducted in Kocaeli, a relatively industrialized city in the northeastern part of Turkey. Eighty-five infants who were referred from the national screening program for congenital hypothyroidism and need venous thyroid function assessment, between February 2010 and August 2010 at Kocaeli University Children's Hospital were evaluated in terms of their vitamin D status as well. This study was approved by Local Ethics Committee of Kocaeli Health Autority (IAEK 3/13 27.10.2009) and conducted in accordance with the guidelines of The Declaration of Helsinki. Written informed consent was obtained from the parents of subjects. (Copies of the written consents are available for review by the Editor-in-Chief of this journal.) The mean age was 263 ± 116 days (84-554 days). All babies had been provided with free vitamin D (Cholecalciferol) solution (Devit-3 oral solution, Deva, Turkey that contains 133 IU vitamin D3 in one drop) and recommended to receive 400 IU (3 drops) daily.

Information regarding the age at start of supplementation, the dosage and compliance were obtained from the mothers with face-to-face interview. The nutrition style and sun exposure were not taken in consideration. Serum 25-hydroxy Intra and inter assay CVs (coefficient variations) were 2.8% and 3.4% respectively. Vitamin D (25-OH-D), alkaline phosphatase (AP), parathormone (PTH) levels were measured. Serum 25-OH-D levels were analyzed and estimated by ELISA reader and microelisa method. Intra and inter assay CVs (coefficient variations) were < 8% and < 10% respectively. Vitamin D status was classified according to Drug and Therapeutics Committee of the Lawson Wilkins Pediatric Endocrine Society (LWEPS) recommendation about cut-off levels for states of vitamin D (25-OH-D< 5 ng/ml: severe deficiency, 5-15 ng/ml: deficiency, 15-20 ng/ml: insufficiency, 20-100 ng/ml: sufficiency) (6). Serum AP level was measured using Abbot Aeroset Autoanalyzer by spectrophotometric method. The manufacturer's normal range for AP was 40-150 U/L, but 145-420 IU/L was accepted normal range for study group (7). Serum iPTH was measured using an original assay using Roche Diagnostics E-170 Modular Analytics immunoanalyzer equipment. The manufacturer's normal range for iPTH was 15-65 ng/L. Intra and inter assay CVs (coefficient variations) were 2.8% and 3.4% respectively.

Statistical analyses were performed using SPSS and student's t test was used for comparison.

## Results

The study group composed of 85 healthy infants (45 girls and 40 boys). The mean birth weight was 3206 ± 53.8 (1370-4680) gr, mean gestational age was 38.8 ± 1.6 (32-42) weeks. The mean age at which Vitamin D3 supplementation began was 16.5 ± 20.7 (3-120) days. Ninety percent of cases (n:76) were receiving 3 drops (400 IU) vitamin D3 per day as recommended; 70% of cases (n:59) were given vitamin D3 regularly, the remainder had imperfect compliance. Among those children who are older than 12 months, only 20% continued vitamin D supplementation (Table 1).

**Table 1:** Demographic characteristics of cases

Number of cases (n)	85
Gender (Male/Female)	45/40
Age (days)	263 ± 116 (84-554)
Birth weight (g)	3206 ± 53.8 (1370-4680)
Gestation age (weeks)	38.8 ± 1.6 (32-42)
Supplementation starting time (days)	16.5 ± 20.7 (3-120)
Cases older than 12 months taking vitamin D	4 (20%)

Seven subjects (8%) were premature. The mean 25-OH-D level of premature infants was 37.7 ± 10.5 ng/ml (21.3-50.4 ng/ml).

No subject had clinical signs of rickets. The mean 25-OH-D level was 42,5 ± 25,8 (median: 38.3) ng/ml. Ten subjects (12%) had their serum 25-OH-D levels lower than 20 ng/ml (6 between 15-20 ng/ml, 3 between 5-15 ng/ml and only one < 5 ng/ml) (Table 2). Ninety-three percent of subjects who reported adequate compliance had 25-OH-D levels within sufficient range (> 20 ng/ml). Twenty seven percent of subjects who had sufficient (> 20 ng/ml) 25-OH-D level had reported inadequate compliance. The mean 25-OH-D level of non-compliant subjects' was 39.6 ± 33 ng/ml (12.9-153 ng/ml). Six out of 10 cases whose 25-OH-D levels were lower than 20 ng/ml reported irregular consumption. All infants with serum 25-OH-D levels lower than 20 ng/ml had normal AP levels, while two had elevated PTH levels. As a group, subjects with serum 25-OH-D levels < 20 ng/ml had significantly higher PTH levels compared with those with vitamin D sufficiency (25-OH-D > 20 ng/ml) (51.6 ± 47.3 pg/ml and 27.2 ± 12 pg/ml respectively, p:0.00). There was no significant difference in AP levels between those two groups (Table 3).

**Table 2:** Vitamin D status of cases

25-OH-D level (ng/ml)	n
< 5 (severe deficiency)	1 (1.2%)
5-15 (deficiency)	3 (3.5%)
15-20 (insufficiency)	6 (7.1%)
20-100 (sufficiency)	71 (83.5%)
> 100 (hypervitaminosis)	4 (4.7%)

**Table 3:** Comparison of cases according to their 25O-H-D levels

	PTH level (pg/ml)	AP level (U/L)
25-OH-D > 20 ng/ml	27.2 ± 12 (9.3-68.6)	249.5 ± 71.4 (119-470)
25-OH-D < 20 ng/ml	51.6 ± 47.3 (13-177)	271.2 ± 48.1 (207-331)
p	0.00	0.35

## Discussion

Vitamin D sources in early infancy consist of transplacental stores, human milk, and cutaneous production via sunlight. High prevalence of maternal vitamin D deficiency, insufficient vitamin D content of human milk, and limited sunlight exposure particularly in the first six months of life all increase risk of vitamin D deficiency and rickets. This is particularly important in infants who are primarily breastfed (8-11). Therefore, daily supplementation of vitamin D appears to be the most efficient strategy to establish adequate vitamin D status and prevent rickets in infancy.

Although there is consensus on the need for vitamin D supplementation, the debate regarding the dose still continues (12). In the past, there has been conflict on the timing and dosage of vitamin D supplementation. European Society for Paediatric Endocrinology (ESPE) Bone Club recommended that all breast-fed infants, regardless of skin color or latitude, should receive 400 IU of supplemental vitamin D per day from birth until they are receiving adequate formula or vitamin D-fortified cow's milk to provide 400 IU of vitamin D per day, in 2002 (13). Nonetheless, American Academy of Pediatrics (AAP) had recommended 200 U/day vitamin D supplementation in 2003 and this recommendation of AAP influenced pediatricians' approach to vitamin D supplementation worldwide (14). Beginning vitamin D supplementation from first month was accepted at the time. However, in geographic areas where maternal vitamin D deficiency is endemic, infantile serum 25-OH-D levels were rickets in breastfed infants in US and other developed decreased < 10 ng/ml earlier than one month (15). Considering the increased incidence of countries, AAP made a new recommendation suggesting 400 U/day vitamin D from the first days of life (6).

Today, it is considered that daily 400 U supplementation of vitamin D is adequate to provide serum 25OH-D level > 12 ng/ml in almost all infants and > 20 ng/ml in the majority of infants (6,11). However, some countries such as Canada continues 800 U vitamin D supplementation per day between December and April (16). There are few studies suggesteing that 400 U is inadequate (17). The results of the current study suggest that daily vitamin D supplementation of 400 U is sufficient to establish serum 25-OH-D levels > 20 ng/ml (vitamin D sufficiency) in great majority of infants if they receive it regularly. It is noteworthy that daily vitamin D supplementation of 400 U appears to prevent rickets even among those infants receiving the supplementation in an irregular manner. We conclude that 400 U/day vitamin D supplementation seems sufficient in Turkey, even in a country where maternal vitamin D deficiency rate is as high as 80% (18-20). These results also support an earlier study reporting dramatic decrease in the incidence of rickets in the Erzurum area following the mentioned program (5). Four infant have 25-OHD levels > 100 ng/ml; Unfortunately we do not have information to explain vitamin D excess. It may be related to vitamin D fortified formula feeding, but we do not know their nutrition type.

Despite strong nationwide strategies continous monitoring of vitamin D intake of infants, i.e. administration via mother is also mandatory. Surprisingly, there is no preventive strategy in United Kingdom where a resurgence of vitamin D deficiency and rickets in the peadiatric population (3). An effective vitamin D supplementation programme does exists in Canada. However, it is reported that 30% of mothers do not give 400 U/day vitamin D on account of feeding their babies with formula (21). Also it is observed that 30% of mothers had not given their babies 400 U/day vitamin D regularly in our study.

In conclusion, we believe that the program for preventing vitamin D deficiency in Turkey, needs to be reinforced to start immediately after birth, and to continue beyond 1 year of age at 400 U regular daily dosage. Nevertheless, other options such as high dose vitamin D supplementations for mothers, particularly during lactation should also be encountered. It is needed more comprehensive studies to conclude that daily 400 U vitamin D is sufficient for term and also preterm babies.

## Competing interests

The authors declare that they have no competing interests.

## Authors' contributions

GYM performed the statistical analysis and interpretation of data and drafted the manuscript, YK participated in the collection of data, EO participated in interpretation of data, FMC and SH participated in the design of the study and final approval of the manuscript. All authors read and approved the final manuscript.
